# Enhanced Security of Bidirectional Communication in IoT-Driven Utility Networks Using Sertainty UXP and LoRaWAN

**DOI:** 10.3390/s26061752

**Published:** 2026-03-10

**Authors:** Zaheen Afroz Simin, Semih Aslan, Marcelo M. Carvalho, Damian Valles

**Affiliations:** Ingram School of Engineering, Texas State University, San Marcos, TX 78666, USA; aslan@txstate.edu (S.A.); mmcarvalho@txstate.edu (M.M.C.); dvalles@txstate.edu (D.V.)

**Keywords:** LoRaWAN, AES, AES-256 GCM, ASCON, XTEA, SPECK, UXP

## Abstract

LoRaWAN holds immense potential in smart applications for its low-power, long-range communication capabilities and in-built AES-128 encryption for end-to-end security. However, prior research has identified critical security vulnerabilities, most notably its use of AES-128 encryption in ECB mode, which lacks semantic security. Sertainty UXP (Unbreakable Exchange Protocol) technology enhances AES by embedding intelligence directly into the data. Sertainty Corporation’s UXP encryption employs AES-256-GCM, which offers authenticated encryption with integrated access control and policy enforcement at the data level, making it a promising candidate for securing sensitive IoT data. The objective of this study is to evaluate whether Sertainty UXP can operate effectively within the strict payload and performance constraints of LoRaWAN. To benchmark performance and overhead, several encryption algorithms, including AES-256-GCM, ASCON-128, SPECK, and XTEA, were implemented for comparison. For experimentation, smart meter data is encrypted with these algorithms and transmitted over LoRaWAN using the LoRa-E5 development board by Seeed Studio. The system’s performance is evaluated based on latency, payload size, and message integrity. Payloads are strategically split into LoRaWAN-compatible chunks and reassembled upon reception to meet network constraints. The results show that integrating UXP encryption within LoRaWAN is technically feasible, though it introduces additional overhead and latency. Despite this, the ability to embed robust encryption and controls directly within the data object offers significant potential to enhance end-to-end IoT security. The research concludes that Sertainty UXP can offer a viable and forward-looking solution for securing resource-constrained networks, provided implementation strategies carefully manage the trade-offs between security strength and transmission efficiency.

## 1. Introduction

### 1.1. Background

In recent years, the Internet of Things (IoT) has dramatically reshaped daily life and multiple industries by enabling real-time communication between vast numbers of interconnected devices. IoT extends the traditional Machine-to-Machine (M2M) communication model by integrating smart devices across residential, industrial, and commercial environments. With the exponential growth of IoT adoption, billions of devices—from home appliances and healthcare sensors to industrial machinery and infrastructure monitoring systems now operate in a unified digital ecosystem. This widespread connectivity enables large-scale communication, but it also creates numerous potential entry points for attackers, thereby expanding the IoT attack surface. For this reason, IoT systems require strong security, as they are widely used in critical sectors such as healthcare, energy, transportation, and smart cities. However, many IoT devices have limited computational power and energy resources, which makes the use of traditional security mechanisms challenging. These challenges are not only theoretical but are also reflected in real-world industry priorities. Around 42% of IT leaders and professionals report that cybersecurity is the primary factor shaping their networking strategies, and this trend indicates that separating networking and security tools, data, platforms, and processes is no longer practical. According to 44% of surveyed organizations, the most significant benefit of unifying networking and security systems is faster detection and response to cybersecurity threats [1]. Prominent researchers, including Butun et al. [2] and Yang et al. [3], have demonstrated that systemic vulnerabilities frequently arise from inadequate authentication protocols, misconfigurations, and the inherent limitations of resource-constrained devices. In this context, long-range, low-power communication technologies such as LoRaWAN are widely used in applications including smart metering, environmental sensing, agricultural automation, and utility monitoring, because they support long-distance communication with low energy consumption. However, as LoRaWAN deployment expands in critical infrastructure, concerns about its security and resilience to evolving cyber threats continue to grow.

According to the LoRa Alliance’s “LoRaWAN Security FAQ” [4], LoRaWAN includes AES-based mechanisms for both confidentiality and message integrity, relying on two primary session keys: the Application Session Key (AppSKey) and the Network Session Key (NwkSKey). While these security measures are conceptually robust, multiple studies have shown that LoRaWAN’s overall security depends heavily on proper implementation, robust key management throughout the lifecycle, and secure backend infrastructure. For instance, Yang et al. [3] showed that LoRaWAN v1.0.2 is vulnerable to replay attacks when using Activation By Personalization (ABP), since its static keys and non-volatile frame counters can be reset or manipulated following a device power cycle. Loukil et al. [5] showed that mixed deployments of LoRaWAN versions 1.0.x and 1.1 can unintentionally reset frame counters during device migration, which can reintroduce replay attack vulnerabilities even after protocol updates. In addition, several backend security issues have been reported in [6], including unencrypted communication between the Network Server(NS) and Application Server (AS). These weaknesses expose critical attack surfaces and increase the risk of rogue gateways, AppKey extraction, and insecure device provisioning. Although Philip et al. [7] reported several security improvements in LoRaWAN v1.1, including root key diversification and bidirectional join counters, they also identified persistent weaknesses in beacon authentication and truncated Class-B counters. Hessel et al. [8] reported real-world deployment issues. These include the use of static AppKeys and unprotected backend APIs, which continue to challenge secure LoRaWAN deployments. Furthermore, the research in [9] demonstrated that reactive jamming can severely degrade LoRaWAN performance. Their results showed significant increases in latency, packet error rates, and energy consumption during interference events, highlighting the vulnerability of LoRaWAN to physical-layer attacks.

### 1.2. Recent Studies on LoRaWAN Security Enhancement

A significant portion of recent work focuses on improving LoRaWAN’s key management and strengthening its cryptographic foundations. Sanchez-Iborra et al. [10] propose the use of Ephemeral Diffie–Hellman Over COSE (EDHOC) as a lightweight authenticated key exchange mechanism, showing it can replace LoRaWAN’s rigid Pre-Shared Key model while fitting strict payload limits. Naoui et al. [11] address similar limitations by introducing reputation-based proxy nodes to support key distribution and by recommending session keys to avoid long-term AES reuse. The research in [12] analyzes several key security models and concludes that, although AES-128 remains practical for constrained devices, LoRaWAN lacks efficient key update procedures and relies too heavily on static secrets. Expanding on the key management landscape, ref. [13] introduced a permissioned blockchain OTAA framework that removes the Join Server as a single point of failure and maintains join records immutably, with performance comparable to ChirpStack. Qadir et al. [14] propose the key generation and distribution (KGD) protocol, combining AES-128 with a secure hash function, Argon2. The proposed protocol uses Elliptic Curve Diffie–Hellman (ECDH) to create shared keys, which helps protect against many security threats. However, because ECDH performs key exchange over an unsecured channel, it can still be exposed to man-in-the-middle (MITM) attacks. To prevent this, the KGD protocol adds the Elliptic Curve Digital Signature Algorithm (ECDSA) so devices can verify each other and ensure the key exchange occurs only between trusted parties. The protocol is then checked with the Scyther tool to test and confirm that the security design works as intended.

To enhance LoRaWAN security, research in [15] suggested that all messages between the NS (Network Server), JS (Join Server), and AS (Application Server) be encrypted, even though this is not mandated by LoRaWAN v1.1. This would prevent payload exposure during backend routing and reduce the risk of man-in-the-middle attacks. Another research study [16] proposes an enhanced OTAA mechanism that encrypts the join request using AES in ECB (Electronic Codebook) mode with the AppKey, ensuring that DevEUI and DevNonce are no longer exposed. Additionally, they introduce a pseudorandom seed (Rseed) and a secure random value (R) to derive a unique AppKey for each session, thereby eliminating the risk associated with AppKey reuse. Eldefrawy et al. [17] perform a formal analysis of LoRaWAN 1.0 and 1.1 using Scyther (a formal security protocol verification tool) and show that v1.0 contains key exchange weaknesses that are resolved in v1.1 under modeled assumptions. Focusing on ABP replay vulnerabilities, ref. [18] introduces a gateway-side replay detection algorithm that blocks replayed packets without disrupting legitimate communication. A gateway compromise study [19] proposes a PKI-based authentication framework in which a two-level certificate authority validates gateway legitimacy, thereby filtering forged gateway traffic. To guarantee security in a dynamic duty cycle, the research in [20] analyzes duty cycle and frame manipulation attacks and proposes protecting join requests using AppKey-based encryption and byte shuffling combined with AES-OCB and hashing to prevent tampering. To ensure end-to-end protection, ref. [21] develops an enhanced LoRaWAN security protocol with default and security-enhanced modes that achieve forward secrecy and replay resistance; the protocol is formally validated with Burrows–Abadi–Needham (BAN) logic and the Automated Validation of Internet Security Protocols and Applications (AVISPA) tool, and is shown to outperform two handshake options, Pre-Shared Key (PSK) and Elliptic Curve Cryptography (ECC), of Datagram Transport Layer Security (DTLS).

Several works investigate LoRaWAN’s physical layer exposure and access authentication performance. For example, Aras et al. [22] demonstrate selective jamming attacks using inexpensive hardware and confirm that LoRa’s slow, robust modulation can be exploited to disrupt specific transmissions reliably. To address server and end device impersonation, a research paper [23] develops a batch authentication scheme that aggregates multiple join procedures using challenge–response and XOR operations, reducing signaling overhead while remaining compliant with LoRaWAN specifications. Other studies explore broader trust, decentralization, and cross-technology comparisons relevant to LoRaWAN’s role in IoT. For instance, Lin et al. [24] integrate LoRaWAN with blockchain to create a decentralized and tamper-proof trust framework for multi-operator IoT service sharing. A comparison study [25] evaluates Mekki major LPWAN technologies—Sigfox, LoRa, and NB-IoT—and concludes that LoRa (and Sigfox) offer superior energy efficiency and coverage for low-rate applications, while NB-IoT targets scenarios needing higher QoS and lower latency.

### 1.3. Encryptions Implemented in Resource-Constrained Environment

Modern IoT utility networks require confidentiality and integrity for telemetry transmitted over constrained links. While LPWAN stacks (e.g., LoRaWAN) provide baseline security at network/application layers, many deployments apply application layer cryptography to protect sensitive payloads end-to-end across gateways, Network Servers, and application backends [4]. A recent LPWAN security survey [26] frames security in resource-constrained environments as a layered system problem rather than a purely cryptographic one. The survey explains that, although LPWAN technologies such as LoRaWAN already employ AES-based protection, security still depends on how confidentiality, integrity, and availability are maintained across end devices, gateways, Network Servers, and backend infrastructure. The study mentions replay-related threats and mitigations such as counter-based replay protection, periodic AppSKey update [27], stronger DevNonce/AppNonce handling [28], and storing previously used DevNonces on the Network Server. It also highlights broader deployment issues including jamming [22], IDS-based jamming detection [29], weak bootstrapping/authentication [30], and untrusted gateways that may record or manipulate data. Taken together, these studies show that practical LPWAN security is not solved by transport encryption alone; it requires complementary protections across the broader system, especially in private industry where intensive security has to be maintained. This provides the motivation of our work: in a resource-constrained environment, additional data-centric security can be studied as a complementary layer that extends protection beyond in-transit cryptography. In this research, we review and evaluate lightweight cryptographic baselines commonly discussed for constrained devices, and separately consider data-centric (object-level) protection, which encapsulates encrypted data along with metadata needed for controlled use [31]. The goal of this review is to motivate the algorithmic choices used in our evaluation and to clarify how each approach influences system overhead in LoRaWAN settings.

Research on securing IoT systems in resource-constrained environments has focused on developing encryption methods that provide strong protection. These methods aim to keep computational, memory, and energy costs low. Several researchers have emphasized lightweight symmetric encryption that is specifically designed for low-power microcontrollers. AbdelRaouf et al. [32] introduced the Muy-Lightweight Cryptographic Algorithm (MLCA), a 64-bit block cipher. This algorithm outperformed earlier lightweight designs such as PRESENT and HIGHT in terms of both energy consumption and speed. Satyanarayana et al. [33] proposed an improved AES variant that adds key whitening and bit shifting steps. This approach achieved lower energy consumption and higher throughput than standard AES on embedded devices. These studies aim to design or refine lightweight encryption algorithms that remain secure while operating within the limited resources of IoT hardware. Satrya et al. [34] evaluated two post-quantum algorithms, NTRU and SABER, on constrained platforms such as Raspberry Pi gateways. Their results showed that both lattice-based methods outperform RSA in terms of speed and resource usage. This finding indicates that quantum-resistant security is feasible for IoT smart-meter applications.

In addition to designing new algorithms, many studies have evaluated existing encryption schemes on realistic IoT platforms to assess their practical performance. For example, Makarenko et al. [35] compared eleven block ciphers using the Contiki-NG operating system. Their results showed that XTEA offers low memory usage and better energy efficiency than PRESENT or SPECK, making it a practical choice for IoT devices. Hasan et al. [36] reviewed lightweight ciphers based on the ISO/IEC 29192 guidelines. [37] They reported that SIMON performs best in hardware implementations, while SPECK shows stronger performance in software. Silva et al. [38] evaluated both symmetric and public key cryptographic schemes on ESP32 nodes. They concluded that AES-128 combined with ECC provides the best balance between speed, power consumption, and code size. Earlier, Khan et al. [39] showed that RSA-1024 and low-bit ECC can operate on Raspberry Pi-based systems. However, these schemes require reduced protocol overhead to remain efficient. So, practical IoT security depends on selecting cryptographic methods that match device limitations, rather than relying on general-purpose algorithms.

Hybrid and layered encryption schemes have also been explored to improve IoT security while meeting energy and timing constraints. Mousavi et al. [40] proposed a hybrid scheme that uses RC4 for fast data encryption. In their approach, ECC is used to secure the RC4 keys, and SHA-256 is added to ensure data integrity. Their method was implemented to resist replay and man-in-the-middle attacks. It showed increased performance compared to common AES–RSA combinations. Another work by Jebri et al. [41] proposed a hybrid approach based on ECC, identity-based encryption, and pseudonyms. This design supports secure and anonymous routing in Raspberry Pi-based IoT networks. It also avoids the certificate-heavy overhead of TLS exchanges. Returning to post-quantum methods, Satrya et al. [34] showed that NTRU [42] and SABER [43] provide strong security with better efficiency than RSA. This result supports the use of modern post-quantum cryptographic methods in IoT-based smart-meter deployments. These hybrid approaches show that combining lightweight symmetric encryption with compact public key methods can provide strong, multi-layered security. At the same time, it remains suitable for devices with limited IoT resources.

Following earlier work on LoRaWAN security and device limitations, different studies have examined how different encryption algorithms perform on low-power IoT devices. A 2024 study [44] showed that AES-256 provides stronger long-term security than AES-128. This improvement comes with only a moderate increase in computational overhead. Based on these findings, Thaenkaew et al. [45] conducted experimental benchmarks on AES-192 and AES-256. Their results showed that the additional processing time is small compared to LoRaWAN radio transmission costs. As a result, larger AES key sizes remain practical for devices operating in low-power wide-area networks (LPWANs). The National Institute of Standards and Technology [46] continues to recommend AES for general-purpose applications. AES in Galois/Counter Mode (AES-GCM) is a common AEAD baseline due to standardization and widespread implementation support. AES-GCM encrypts using counter mode and authenticates using a polynomial hash (GHASH). Here, nonce uniqueness is critical, because nonce reuse can compromise confidentiality and integrity [47]. In constrained IoT contexts, AES-GCM is often treated as a “conventional” reference point: it is robust and well-supported, but its performance and packet-format overhead depend on nonce size, authentication tag and implementation environment.

At the same time, NIST [46] has identified ASCON as the preferred lightweight authenticated encryption with associated data (AEAD) algorithm for highly constrained environments. ASCON [48] uses a sponge-based permutation design and requires minimal RAM. It also provides built-in authentication, which has supported its use in many IoT research prototypes. Ascon-128 provides authenticated encryption with a nonce and authentication tag, and it is frequently considered when designers need AEAD security with reduced implementation footprint compared to more traditional constructions. On the other hand, lightweight block ciphers such as SPECK and XTEA have very short encryption and decryption times, often measured in tens of microseconds. SPECK is a family of lightweight ARX-based block ciphers designed for efficient implementation in resource-limited environments [49]. XTEA (eXtended TEA) is another compact block cipher designed for simplicity, commonly used as a lightweight baseline in embedded contexts. It is a 64-bit block cipher based on a Feistel network and uses a 128-bit key. It was introduced to address known weaknesses in TEA and was developed by David Wheeler and Roger Needham at the Cambridge Computer Laboratory. The algorithm was first reported in [50]. It is an attractive algorithm for latency-critical and energy-limited scenarios. Unlike AEAD schemes, a block cipher alone does not inherently provide message authenticity; integrity protection typically requires a separate MAC or an authenticated mode of operation. Also, these algorithms provide lower security margins than more robust AEAD schemes.

In addition to algorithmic encryption, data-centric security standards have been developed to address the limitations of transport- and channel-based security approaches. Modern utility systems require more than just encryption; they need intelligent data protection beyond traditional encryption methods. LoRaWAN security implementation can be complex and sometimes it includes third party software which might compromise security. This is where Sertainty UXP technology [51] enters the picture. UXP technology strengthens an organization’s existing security layers by embedding access control intelligence and protection policies directly into the data. By doing so, it protects the entire data lifecycle from creation to expiration or destruction. It is a data-centric protection approach that converts an unstructured dataset into a self-protecting binary called a UXP Object (file extension *.uxp). The protection mechanism combines AES-256-GCM encryption with additional proprietary methods that eliminate the need for a separate key-sharing step [52]. Keys are generated during the conversion process and managed internally within the UXP Object. The resulting intelligence and protection are embedded directly into the dataset. They are packaged invisibly inside a UXP Object that moves as a single unit—appearing as an inert binary file that can be interpreted only by UXP technology libraries. Although it internally uses AES-256-GCM as part of its protection scheme, it is not equivalent to applying AES-256-GCM directly to an LoRaWAN payload. Instead, UXP “objectizes” the data by blending the dataset with UXP metadata and enforcement components so that access control and governance can travel with the data across storage, transmission, and downstream processing. Because Sertainty’s implementation includes proprietary mechanisms and the source code is not available to us, we describe UXP using vendor technical documentation and evaluate its impact as a black box workflow in our LoRaWAN testbed. Also, its performance in low-power wide-area network (LPWAN) environments, such as LoRaWAN, has not yet been evaluated. There are limited empirical data on UXP’s processing overhead, transmission impact, and sensitivity to environmental conditions. This gap highlights the need to assess UXP’s suitability for secure communication in LPWAN deployments.

Based on the above, this work selects (i) AES-GCM as a conventional AEAD reference, (ii) Ascon-128 as a modern lightweight AEAD reference, and (iii) SPECK and XTEA as compact block cipher baselines, to cover a representative spectrum of algorithmic designs used or discussed for constrained devices. We then compare these against a data-centric object workflow (UXP) to quantify end-to-end system overhead across LoRaWAN probe points. This scope keeps the analysis centered on measurable latency/packet handling impact rather than product-level claims.

### 1.4. LoRaWAN Performance in Prior Studies

LoRaWAN performance is strongly influenced by environmental and propagation conditions, a conclusion supported by a wide range of studies. Research in [53,54] found that signal strength and reliability are significantly lower in indoor or obstructed environments than in outdoor, line-of-sight (LOS) scenarios. Research by Muzammir et al. [55] demonstrated that deploying multiple gateways can help counteract signal attenuation and improve reliability in dense indoor settings. Addressing LoRaWAN’s limited data rates and strict duty cycle regulations, Marini et al. [56] propose a hybrid multi-band, multi-hop LoRaWAN design to balance the high data rates of 2.4 GHz with the long-range benefits of sub-GHz links. Using their open-source, standard-compliant, proprietary LoRaWAN simulation framework [57], they evaluated the architecture. The architecture outperforms standard single-band LoRaWAN, offering a practical path toward more scalable next-generation IoT networks with minimal modification of the core architecture. Magrin et al. [58] further quantified how factors such as spreading factor selection, confirmed message usage, and gateway placement affect LoRaWAN’s packet delivery rate, airtime, and energy consumption. Research in [59] showed that appropriately optimized radio configurations can preserve reliability while reducing energy consumption under favorable conditions. Outdoor studies [60,61] indicated that rural LOS (line-of-sight) environments support multi-kilometer communications. In contrast, urban and suburban NLOS (non-line-of-sight) conditions exhibit pronounced variability due to multipath fading, shadowing, and fluctuating signal-to-noise ratios (SNR).

Application-specific deployments—such as Chaudhari et al.’s [62] manhole monitoring framework and Qazi et al.’s [63] secure, encrypted underground utility monitoring system—not only demonstrate LoRaWAN’s versatility but also point to the persistent lack of standardized methods for secure, device-level protection.LoRaWAN deployment in smart campus environments has recently emerged as an increasingly popular. Pasetti et al. [64] and Sánchez Sutil et al. [65] evaluated LoRaWAN performance in university settings, testing various message intervals, payload sizes, and gateway placements. These studies report high packet delivery ratios in outdoor line-of-sight contexts, but note performance degradation in indoor or obstructed layouts, particularly with low spreading factors. Lozano et al. [66] conducted a well-rounded study that compares indoor and outdoor perspectives by thoroughly testing environmental effects and physical obstructions. Their findings show that higher spreading factors improve robustness but extend airtime, and wall materials significantly affect indoor communication. Importantly, their integration of RSSI and PDR across settings assessed overall link quality and system performance.

### 1.5. Research Gap and Motivation

Despite the breadth of prior research, several gaps remain unaddressed. No study has conducted a comparative analysis of multiple encryption algorithms, such as AES-256-GCM, ASCON-128, SPECK, and XTEA, within the same LoRaWAN deployment to assess how encryption overhead influences communication latency directly. Additionally, existing research has not broken down end-to-end latency into the individual LoRaWAN segments, including an end device to gateway, gateway to Network Server, and Network Server to Application Server under different cryptographic loads. Although lightweight cryptography and authenticated encryption with associated data (AEAD) schemes have been studied separately, their combined effects within environmentally sensitive LoRaWAN deployments remain unexplored. Most notably, Sertainty’s UXP framework has not been evaluated in LoRaWAN or any low-power wide-area network (LPWAN) context, leaving a significant gap in understanding the practicality of data-centric security models in long-range, low-power communication environments.

Motivated by these gaps, this research presents the first comprehensive experimental analysis of five encryption schemes, such as AES-256-GCM, ASCON-128, SPECK, XTEA, and Sertainty UXP, alongside an unencrypted baseline, measuring their impact on LoRaWAN uplink latency across multiple distances and environmental conditions. By collecting synchronized timestamps at the end device, gateway, Network Server, and Application Server, the study decomposes latency into its core components. It quantifies how cryptographic overhead interacts with propagation effects and backend processing in a real LoRaWAN deployment. In addition, this work investigates how secure bidirectional communication can be achieved in IoT-driven utility networks by integrating Sertainty UXP with a practical LoRaWAN testbed. The system includes a Class an LoRaWAN end device, a commercial gateway, a cloud-based Network Server, and an EC2-hosted Application Server that embeds UXP-protected objects into LoRaWAN payloads and evaluates them under real channel conditions and distances. Within this framework, UXP-protected traffic is systematically compared against conventional LoRaWAN security and other encryption approaches to examine differences in payload size, link reliability, latency, and transmission range. Together, these contributions address the current lack of performance assessments for end device encryption in resource-constrained LoRaWAN systems and evaluate the practical feasibility, overhead, and configuration trade-offs of adopting Sertainty UXP as a data-centric security layer for future critical IoT and utility applications.

## 2. Materials and Methods

### 2.1. Overview of LoRaWAN Architecture and Security

**Figure 1:**
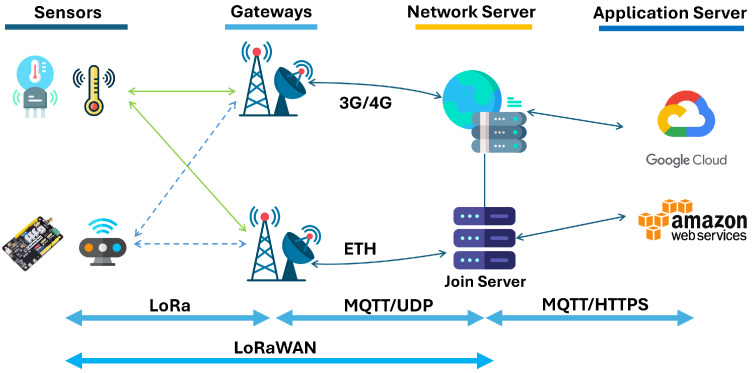
LoRaWAN architecture.

Figure 1 shows the basic architecture of LoRaWAN. At the edge, end devices generate low-power wireless data; this data is forwarded by gateways using IP backhaul; the Network Server then performs routing, de-duplication, and MAC layer management; the Join Server handles secure device activation and session-key generation; and finally, the Application Server (AS) processes decrypted payloads and delivers them to cloud platforms for analytics and visualization. More details about this architecture are given below.

In theory, LoRaWAN is a MAC protocol layered on top of the LoRa PHY, operating primarily in the sub-GHz ISM bands (e.g., US915 and EU868). End devices transmit using chirp spread spectrum (CSS) with configurable spreading factors (SF7–SF12), which determine the time-on-air, link budget, and achievable data rates. Devices operate in Class A by default, which initiates all communication via uplink transmissions and opens deterministic downlink reception windows (RX1 and RX2) after each uplink transmission. MAC commands (e.g., LinkADRReq and RXParamSetupAns) may be embedded in either the FOpts field or FRMPayload, depending on encryption requirements. On the other hand, Class B devices add scheduled receive windows synchronized through beacon signals, enabling more predictable downlink access. Class C devices keep their receive window open almost continuously, providing the lowest latency at the cost of higher energy usage. This research utilizes a Class A end device, which offers ultra-low-power operation suitable for battery-constrained IoT deployments.

In Class A, uplink frames are broadcast omnidirectionally, allowing all gateways within RF range to demodulate the signal concurrently. Gateways implement a multi-SF, multi-channel front-end and forward demodulated packets to the Network Server through the Semtech UDP Packet Forwarder or LNS-compliant BasicStation protocol. Gateways do not interpret frames; they encapsulate metadata (RSSI, SNR, timestamp, and CRC status) and deliver it upstream.

The Network Server performs MAC layer intelligence and integrity control. It executes packet deduplication (based on DevAddr, FCntUp, and MIC), verifies message integrity using AES-CMAC with the NwkSKey, enforces frame counter synchronization, and manages the Adaptive Data Rate (ADR) algorithm by optimizing SF, TX power, and channel mask configurations. The Network Server also handles downlink queuing, strictly complying with regional duty cycle limits, and elects appropriate gateways for transmission based on link margin and availability.

**Figure 2:**
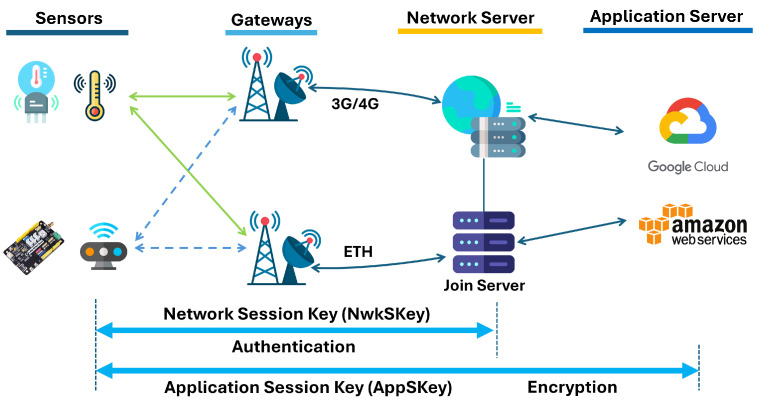
Two layers of security in LoRaWAN.

After validation, the Network Server forwards the FRMPayload to the Application Server (AS) without decrypting it. At the AS, the payload is decrypted using the AppSKey via AES-128 in CTR mode. This cryptographic separation is fundamental to LoRaWAN’s dual-layer security model: NwkSKey secures network layer integrity, while AppSKey enforces end-to-end confidentiality. When the Network Server and Application Server are co-located, as seen in many commercial deployments, this separation may be violated, making a standalone AS (e.g., on AWS EC2) beneficial to preserve formal LoRaWAN security guarantees. Figure 2 shows two layers of security in LoRaWAN.

**Figure 3:**
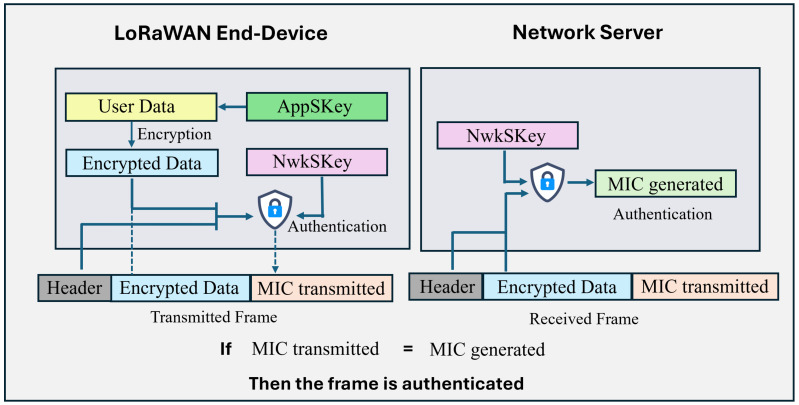
Device authentication by network server.

To ensure secure communication within an LoRaWAN network, several security measures have been incorporated into its design. For instance, the Network Session Key (NwkSKey) is generated when an end device connects to the network. This is a 128-bit AES key used to authenticate the payload transmitted from the end device to the Network Server. The NwkSKey is utilized to authenticate communication between the LoRaWAN end device and the Network Server. To enable this authentication, a Message Integrity Code (MIC) is appended to each frame. The MIC is generated based on the encrypted payload and the NwkSKey. Upon receiving the message, the Network Server performs the same MIC calculation. Suppose the NwkSKey stored on both the end device and the Network Server matches. In that case, the MIC generated during the reception will align with the MIC attached to the transmitted frame, confirming the message’s authenticity. The authentication process occurs between the end device and the Network Server. Once authentication is successful, the Network Server forwards the message to the Application Server. Figure 3 shows how the user data is authenticated in the Network Server.

At this stage, encryption becomes critical. The Application Session Key (AppSKey), another 128-bit AES key, provides end-to-end security from the end device to the Application Server. In summary, the NwkSKey ensures payload authentication between the end device and the Network Server, while the AppSKey ensures encryption between the end device and the Application Server. End-to-end security in LoRaWAN refers to the ability to encrypt user data at the end device and decrypt it only in the user application. Table 1 shows the key difference between AppSKey and NwkSKey.

However, achieving this level of security is not always straightforward, especially when different entities host the Network Server and Application Server. Another concern is that the NwkSKey and AppSKey are stored on the end device, making them vulnerable to side channel attacks. If the system is not correctly implemented, decrypted payloads may become visible on the Network Server, compromising end-to-end confidentiality. These challenges underscore the need for robust implementation and secure key management practices in LoRaWAN networks.

### 2.2. Sertainty UXP Data-Centric Security Framework

This research applies Sertainty Corporation’s [67] UXP technology within LoRaWAN. This section provides an overview of this technology, its key concepts, and its potential for the future cybersecurity industry. Sertainty provides a data-centric security framework that places protection directly inside the data. This design removes the need for external perimeter controls, network boundaries, or host-based defenses. Instead, the data enforces its own rules wherever it moves. The foundation of this approach is the Intelligent Cipher Transfer Object (ICTO) [68], which functions as a self-protective data unit that contains encryption, access rules, and authentication logic within a single object.

**Figure 4:**
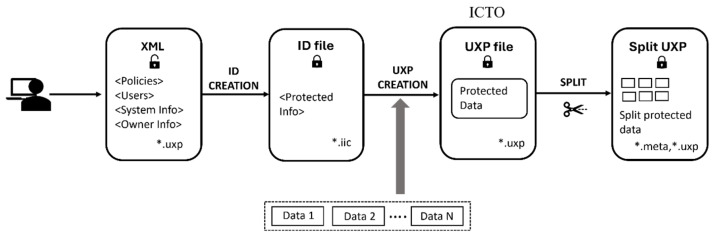
ICTO Object creation.

The ICTO is created from an XML file that defines the complete set of security parameters. These include user identities, allowed operations, access conditions, authentication requirements, and optional environmental constraints. This XML is processed into a secure *.iic ID file that stores all rules in encrypted, immutable form. When the ID file is combined with the actual user data, the system generates the ICTO Object. During creation, encryption such as AES-256-GCM is applied to maintain confidentiality and detect tampering. The resulting object can evaluate access attempts, enforce owner-defined rules, log activity, and block or destroy itself if unauthorized interaction occurs. The ICTO can also be safely split into multiple components and later recombined without losing its embedded controls, increasing its flexibility in distributed systems. Figure 4 shows the step-by-step process of ICTO creation.

Sertainty’s Unbreakable Exchange Protocol (UXP) extends the ICTO model into a complete protection system for unstructured data. UXP works with files such as text documents, spreadsheets, images, CSVs, and videos. Through UXP, these files become UXP Objects, which carry encrypted content, device-specific fingerprints, and an internal rule execution engine. This engine is implemented as the Kernel Control Language (KCL) Program (Proprietary language of Sertainty), which manages encryption keys, enforces the rules set, and performs authentication at the moment of access.

A UXP Object is governed by a UXP Identity (UXP ID): a protected *.iic file similar in concept to the ICTO ID file. The UXP ID defines trusted devices, allowed time windows, geographic limits, accepted users, and failure-handling policies. During object creation, the UXP technology verifies the UXP ID and embeds its rule set directly into the object’s metadata. Once created, the object appears inert, unreadable, and structurally indistinguishable from an opaque binary file.

When the object is placed into an environment containing UXP technology libraries, the embedded KCL Program activates. It checks the runtime context—including the machine fingerprint, user identity, network properties, geographic location, and time of access before decrypting any content. Access is granted only if all the conditions match the original rule set. Otherwise, the object denies access silently or executes a defined protective response. This ensures that data remains protected even when stored on untrusted devices or transmitted through insecure networks.

Each UXP Object is cryptographically unique because the system uses randomized key generation and individualized metadata. Importantly, encryption keys are generated internally and remain hidden within the object, eliminating the need for key sharing, storage, or external key management. UXP Objects may also maintain tamper-proof event logs that record both valid and invalid access attempts, supporting auditing and incident analysis without exposing the file’s internal structure. AES-256-GCM payload encryption typically means encrypting a bounded application payload and then sending that payload through the network. UXP differs because encryption is only one part of a larger object-based workflow that also includes (i) embedding rules/policy artifacts, (ii) an internal enforcement program, and (iii) object metadata structures required for activation, authentication, and optional auditing. Therefore, UXP overhead should be expected to appear primarily through object formation and object handling steps, not only as per-packet cipher time.

In summary, both ICTO and UXP apply the same core principle: data carries its own security. By embedding encryption, authentication rules, and contextual access control directly into the data object, Sertainty provides persistent and portable protection across diverse environments. This supports strong, consistent, and autonomous security for sensitive unstructured information.

### 2.3. Secure Data Processing Pipeline Using Sertainty Data Protector Utility

The Sertainty Data Protector Utility is a dedicated tool built to support the complete lifecycle of UXP-based data protection. It serves as an operational interface for applying UXP technology without requiring direct access to the underlying SDK. The utility manages key activities such as protection, splitting, joining, and the unprotection of data. It internally relies on the Sertainty UXP Engine to carry out all cryptographic and policy-embedding operations. When a task is executed, the utility performs several automated steps, including AES-256-GCM encryption, cloaking through Sertainty’s proprietary algorithms, embedding UXP access rules, integrating the KCL Program, and generating tamper-evident signatures. These steps ensure that data remains self-protecting during storage, transmission, and access.

**Figure 5:**
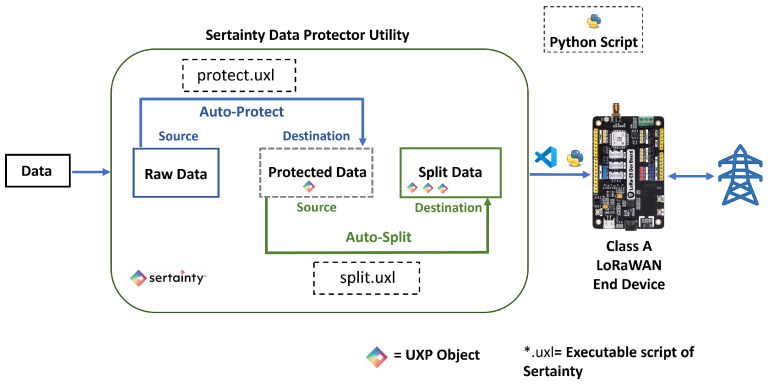
Workflow of the transmission side of Sertainty Data Protector Utility.

**Figure 6:**
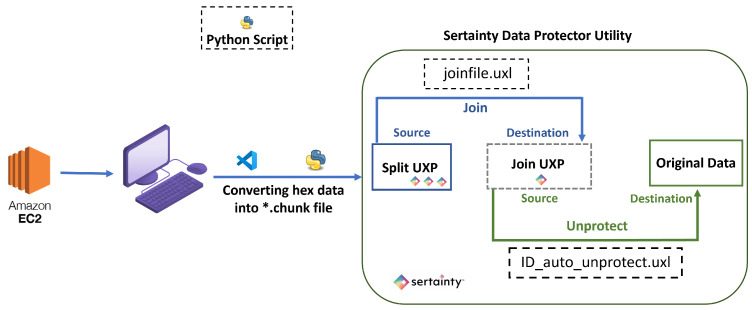
Workflow of the receiver side of Sertainty Data Protector Utility.

Sertainty Data Protector Utility is used to perform encryption and decryption. It follows a systematic workflow for encrypting, transmitting, and decrypting data. Encryption and splitting are performed prior to dispatching the data on the LoRaWAN architecture. After the data reaches the Application Server (AS), the data is extracted from the AS, and it is decrypted using the Sertainty Data Protector Utility. Below is a detailed breakdown of the workflow:

- XML and ID File Creation: The utility first generates an XML file to encrypt the data. This file holds the security policies, user access controls, and system definitions. It specifies who can access the data and under what circumstances. The XML file is a blueprint for securing the data, and it is used to create an ID file (*.iic), which stores all the access control policies and authentication parameters needed to secure the data.
- Configuration and Task: After creating a machine ID, the user can create several configurations, each containing multiple tasks under that machine ID. Each task includes a pre-built script containing essential information required to process the data and convert it into the UXP format specified by the user. Figure 5 shows two tasks, “Auto-Protect” and “Auto-Split,” on the transmission side.
- Setting up Tasks: After creating a machine ID, tasks are configured using executable scripts that define key parameters, such as the source files, destination folder, ID file, delete flag, event ID, and optional alternate names for UXP files. Each executable verifies the event UXP file, generates unique output filenames using timestamps, and appends a “multi” suffix when multiple files are protected. The process is logged in an XML document recording start time, destination, ID, events, and delete settings. If UXP creation fails, the error is logged, and the process stops; if the delete flag is set to “true,” original files are removed after protection. The “Split.uxl” executable divides files into multiple chunks based on size or count for optimized transfer, creating unique metadata (*.meta) and split (*.uxp) files with timestamps. Each step and outcome is recorded in an XML log, enabling traceability. Once tasks are defined, the Data Protector Utility executes the operation, specifying the source, destination, and chunk size.
- Auto-Protect and Auto-Split: In the “Auto-Protect” task, preprocessed smart meter data is continuously monitored and encrypted into *uxp files using the Sertainty Data Protector Utility from the source folder to the destination folder. The resulting encrypted files trigger the “Auto-Split” task, which divides them into smaller encrypted chunks (minimum 50 bytes) with corresponding metadata for reassembly. For the “Auto-Split” task, the source folder is where the earlier task left its output (*.uxp), and, in the destination folder, we get the split version of the *.uxp files (shown as *.chunk extension) with a corresponding *.meta file (shown in Figure 6). These chunks are then sent to the LoRaWAN transceiver for efficient, secure transmission under bandwidth constraints. A Python (version 3.11.9) observer script coordinates and logs each stage, measuring task completion times to ensure smooth operation.
- Join and Decryption: After receiving the split UXP files, the Sertainty Data Protector Utility joins and decrypts them under the same machine ID. Before that happens, the data is extracted from the EC2 instance in the cloud and converted back into *.chunk file with the help of the *.meta file produced on the transmission side to successfully join all the split files sent over LoRaWAN. Once all payload chunks are received and converted, they are accumulated in the source folder where the “Join” task will take place. The *.meta file triggers the Join task, and it reconstructs the original data. Two executables are used: Joinfile.uxl, which merges split files into complete originals, assigns unique filenames, appends suffixes if specified, logs all operations in XML and deletes split files upon completion, and ID_auto_unprotect.uxc, which automatically decrypts files using an auto SSO session linked to the user’s *.iic file. This script removes protection, saves the decrypted outputs, and logs all actions without requiring manual authentication, ensuring secure and streamlined data restoration. So, all of these are integrated with the main setup, and everything is monitored and calculated at different distances from point A of the experimental setup.

**Figure 7:**
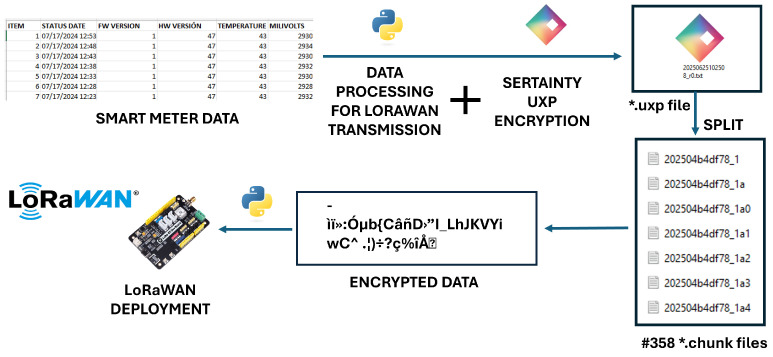
Different stages of smart meter data conversion using Sertainty encryption.

Figure 7 shows how the data looks at different stages in Sertainty UXP processing before deploying it to the LoRaWAN network. The process begins with raw data extracted from the smart meter at the STAR Park facility. A Python script preprocesses this data, isolating key parameters such as firmware version, hardware version, temperature, and millivolts. The preprocessed dataset is then passed to the Sertainty Data Protector Utility, which encrypts it into a secure UXP Object (e.g., 20250625102508_r0.txt). The script extracts and preprocesses the data row by row at a certain interval indicated in the script to mimic real-time data extraction. Once the encrypted file is generated, it is processed through the “Auto-Split” task, which divides the UXP file into multiple smaller encrypted chunks (e.g., 202504b4df78_1a0, 1a1, etc.) to comply with LoRaWAN’s low data rate and payload limitations. Each chunk retains the same level of encryption and is accompanied by metadata necessary for reassembly. Another Python observer script then sends these encrypted chunks through the LoRa-E5 transceiver, transmitting them over the LoRaWAN stack for secure and efficient long-range communication.

### 2.4. Experimental Setup

**Figure 8:**
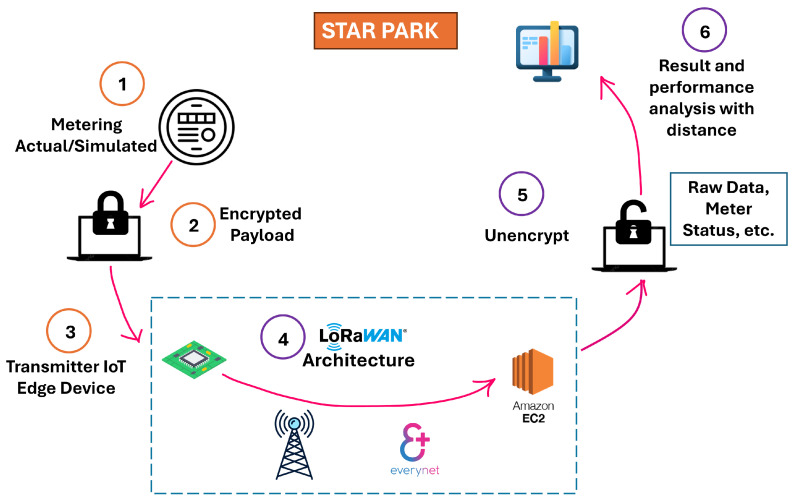
Overall setup of one-way transmission.

This section describes the methodology used in this research. It includes the implementation of the LoRaWAN architecture and the integration of Sertainty UXP technology with the data prior to its deployment within the LoRaWAN network. The Sertainty Data Protector Utility, as described in the previous subsection, plays the main role, from data handling to the encryption and decryption of the Easymetering smart meter data [69]. Easymetering provides advanced smart metering solutions built around cellular and private-LTE-enabled Smart NIC modules, offering interoperable AMI connectivity, real-time meter data acquisition, and cloud-based analytics compatible with ANSI and DLMS/COSEM standards. The setup begins with data extraction from a smart meter located at the STAR Park facility at Texas State University. The smart meter stayed offline during the writing of this article for maintenance. All the experiments are conducted using data previously collected while the smart meter was online. This raw data is filtered to isolate the critical parameters necessary for transmission and sent over the LoRaWAN network periodically at a consistent interval to mimic real-time data transmission. The filtered data is then encrypted into the UXP Object using the Sertainty Data Protector Utility, ensuring that sensitive information is secured against unauthorized access. Figure 8 illustrates the overall process of the experimental setup.

To comply with LoRaWAN’s low data rate limitations, the encrypted data is split into multiple smaller segments, which allows it to be transmitted within the permissible payload of the LoRaWAN system. For other encryption and raw data transmission over LoRaWAN, the data is not required to be split due to the very low overhead. To transmit UXP-protected data over LoRaWAN, the workflow involves object creation followed by Split/Join-style fragmentation and reassembly to fit LoRaWAN payload limits. The SDK datasheet document of Sertainty main repository [52] describes Split/Join as a core module used with object construction and workflow scripting. As a result, the UXP overhead in a constrained LPWAN is expected to be dominated by (i) object generation and splitting before transmission, (ii) the increased number of frames due to fragmentation, and (iii) reassembly and object processing after reception. This differs from AES-256-GCM payload encryption, where the dominant added cost is encrypt/decrypt computation on a fixed payload. These encrypted segments are passed to a LoRa-E5 development board (end device). As shown in Figure 8, after preprocessing the encrypted data generated by Sertainty UXP technology, it is transmitted through the LoRaWAN architecture, beginning with the end device, followed by the gateway, the Network Server (Everynet) and finally the Application Server (Amazon EC2 instance). Once received, the data is decrypted on a separate machine, where system analysis is performed.

There are five data rate (DR) settings in LoRaWAN (DR0–DR4). These can be selected manually during device configuration, or the end device can determine the most suitable DR based on the network’s performance. This research uses the DR3 setting (Spreading Factor 7 and Bandwidth 125 kHz), as it provides a practical balance between the communication range and the available payload size. To fit the transmitted information within the payload limitation of DR3, only the first letter of each parameter name and its corresponding value were extracted and sent for retrieval on the receiver side.

To establish the LoRaWAN architecture (Part 4 of the setup), specific hardware and software components were selected. The Wio-E5 development board [70] by Seeed Studio is chosen as the end device. This board features the Wio-E5 STM32WLE5JC module, a system-on-chip that integrates a Semtech SX126x LoRa transceiver with an ARM Cortex-M4-based STM32 microcontroller(Seeed Studio, Shenzhen, China). Within the Cortex-M family, the M3 and M4 models provide an effective balance between computational performance and energy efficiency. The module supports LoRa and LoRaWAN protocols across multiple global frequency bands, including EU868, US915, AU915, AS923, KR920, and IN865.

For the gateway, a TEKTELIC KONA Mega gateway [71] is used and installed on the rooftop of the STAR Park facility at Texas State University. This gateway supports full-duplex communication across multiple spreading factors and operates over several ISM bands, enabling wide-area and multi-region LoRaWAN coverage.

Everynet [72] (currently owned by Netmore) is utilized as the Network Server. It is a central component in LoRaWAN deployments, offering a scalable and flexible platform that manages the routing, filtering, and delivery of LoRaWAN messages. When end devices transmit uplinks to nearby gateways, the gateways forward the data to the Everynet Network Server, where all core processing and message handling occur. Everynet [73] provides two primary external interfaces: a RESTful API and a Streaming API. The RESTful API is used for device provisioning, configuring network rules, and managing Application Server (AS) connections. The Streaming API, implemented using WebSocket, supports the real-time delivery of uplink and downlink messages, which is useful for low-latency IoT applications and real-time debugging. In addition, Everynet offers built-in adapters for protocols such as MQTT and HTTP, simplifying third-party integration without requiring custom middleware. In this configuration, Everynet provides the Network Server component of the LoRaWAN stack, while a third-party AS must be deployed separately.

The end device (ED) connects to the Network Server through the gateway using Over-the-Air Activation (OTAA). After registering the Device EUI, Application EUI, and other required parameters as described in the LoRa-E5 documentation [70], the device joins the network using an AT command issued through a serial interface.

The Application Server layer employed in this work is a small Ubuntu 22.04 instance running on AWS EC2 [74] (t2.micro free tier). Its configuration is intentionally minimal. A single program, the Mosquitto MQTT broker (version 2.0), listens on port 1883 and accepts connections only from Everynet’s Network Server, whose IP range is whitelisted in the instance’s security group. When an end device transmits an uplink, the nearest gateway forwards the LoRaWAN frame to Everynet; the Network Server authenticates the message with the Network Session Key and then pushes the still-encrypted MAC payload to the broker on the VPS via the topic/everynet/uplink LoRaWAN NS.

The traffic is not decrypted inside the virtual machine because the Application Session Key (AppSKey) has not been deliberately installed. Instead, a lightweight subscriber running from the command line mosquitto_sub-t/everynet/uplink prints each base-64 string exactly as received. The VPS, therefore, acts as a cryptographic “airlock” and provides a place under the researcher’s sole control where the ciphertext can be observed, time-stamped, and archived without exposing the plaintext to the network operator or the public internet. Because no decryption is attempted, the compromise of this machine would reveal nothing but AES-128 cipher-text, so the end-to-end confidentiality promised by LoRaWAN remains intact.

**Figure 9:**
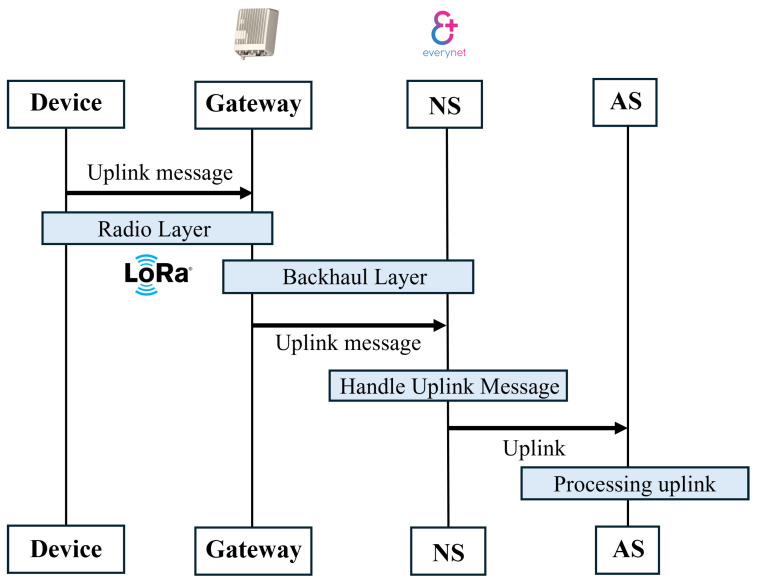
Sequence diagram of how uplink messages work.

A virtual private server (VPS) environment is set up to enable communication between the LoRaWAN Network Server (NS) provided by Everynet and a custom-built Application Server (AS). For this purpose, Amazon Web Services (AWS) Elastic Compute Cloud (EC2) is chosen due to its scalability, reliability, and accessibility within the AWS Free Tier. After successfully setting the Application Server (AS), the architecture is complete. The Application Server and Network Server communicate in a certain way, which makes the bidirectional communication in LoRaWAN possible. Figure 9 shows how an uplink (from the end device to the AS) works. In the uplink message delivery process to the Application Server (AS), the device first sends a message to the gateway. The gateway processes the message and adds extra information, such as radio frequency (RF) parameters, before forwarding it to the Network Server. The Network Server receives the message, performs operations like decryption and deserialization, and then sends the processed data to the AS using the Data API. Finally, the AS receives the message, which is marked with the type “uplink”.

After the technical setup was completed, the required scripts were developed, and all experiments were conducted at the STAR Park research center. The objective of the experiment was to evaluate the performance of several encryption standards that are commonly used in resource-constrained environments and to assess their impact on latency across different nodes in the LoRaWAN transmission architecture. The selected encryption methods include (1) ASCON, (2) XTEA, (3) AES-256, (4) SPECK, and (5) Sertainty UXP encryption.

Although a significant amount of research has been conducted on LoRaWAN, very few studies have examined the impact of applying additional encryption layers on top of LoRaWAN’s built-in security mechanisms. Specifically, the latency introduced at each stage of the data transmission process has not been extensively explored.

**Figure 10:**
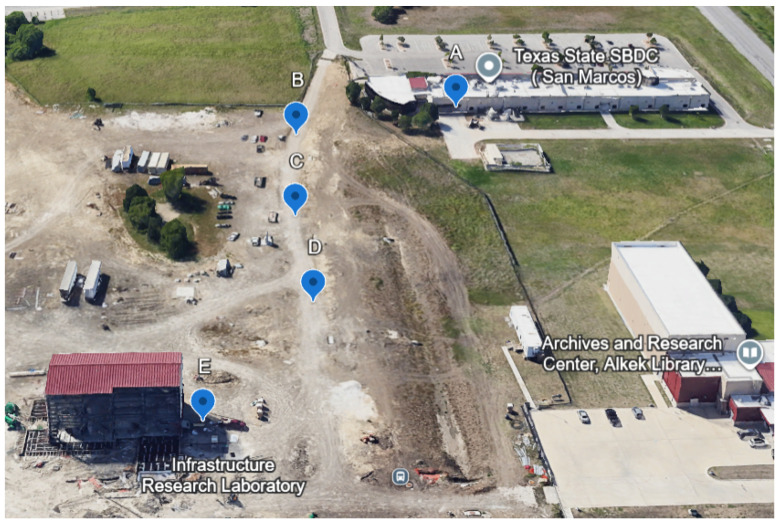
Experimental test-point locations at STAR park.

Figure 10 presents the points from where the tests were conducted. The 0 m distance represents testing conducted inside the building below the gateway on the roof, while the remaining distances represent outdoor testing. For each encryption method, 100 payloads were transmitted to the gateway located on the building’s rooftop. Timestamps were recorded at four key points: the start of transmission at the end device, the time the signal was received at the gateway (rx_time), the time it was processed by the Network Server (meta_time), and the time it reached the Application Server. The values for rx_time and meta_time were obtained from the JSON log files available through the Everynet Network Server portal. The definitions of these timestamps are provided in the Everynet Network Server documentation [73]. The test sites were selected along the paved road in front of STAR Park. The screenshot, taken from Google Earth, does not reflect the most recent satellite imagery. Currently, the area appears as an open grass field. Due to accessibility issues, the pavement between the STAR Conference Center and the IRL building was chosen for testing. All subsequent distance measurements were taken from the 0 m reference point (Point A) (Figure 10). There are, in total, five points where the tests have been conducted (A, B, C, D, and E). A indicates zero points and B is 50 m from point A. Similarly, points C, D, and E represent distances of 100, 150, and 200 m from the reference point.

Point A is located inside the Communication Lab of the STAR Conference Building at Texas State’s CIEDAR facility. This location experiences the highest level of interference due to the presence of multiple active communication technologies within the lab, and the thick surrounding walls further obstruct the signal. The 50 m point (B), although close to the building, does not provide a clear line of sight (LOS); trees and partial structural obstructions lie between the end device and the gateway. In contrast, the 100 m (C) and 150 m (D) points offer optimal conditions, with clear LOS and minimal interference, making them ideal for signal transmission tests. The 200 m point (E) is positioned just outside the IRL building. While it mostly offers direct visibility, slight obstructions such as nearby trees and walls may affect signal strength. Latency measurements showed convergence beyond 100 m under line-of-sight conditions, with no significant variation observed up to 200 m. As a result, the experiments were concluded once stable behavior was confirmed. A spot check at 250 m showed the same stable latency behavior observed between 100–200 m. Therefore, this study reports results up to 200 m, since the key distance-dependent transition and fluctuations were already captured within the 0–200 m range. Longer-distance measurements have potential to provide additional insight in latency behavior over extended links and will be considered in future work.

### 2.5. Latency Calculation of Different LoRaWAN Probe Points

To accurately measure latency across the LoRaWAN transmission path, all scripts were designed to capture critical timing variables associated with different network stages. Four synchronized timestamps were used to quantify different probe points of transmission latency.

- send_time: The exact moment when the payload is dispatched from the end device via the LoRa-E5 transceiver.
- rx_time: The time recorded by the gateway when the transmitted signal is received from the end device.
- meta_time: The timestamp generated by the Network Server when the gateway forwards the message upstream for network processing.
- ec2_publish: The moment when the Application Server (AS), hosted on the Amazon EC2 instance, receives and logs the payload from the Network Server through the MQTT broker.

The Python script is used only for payload preparation (formatting/encoding) prior to transmission; since send_time is captured at dispatch via the LoRa-E5, the reported LoRaWAN probe point latencies do not include this host-side preparation time. All timestamps are recorded in Unix epoch format (milliseconds), ensuring a consistent temporal reference across devices operating in different environments and time zones. These synchronized logs allow precise computation of three latency components:

- End device to gateway (rx_time − send_time),
- Gateway to NS (meta_time − rx_time),
- NS to AS (ec2_publish − meta_time)

### 2.6. Implementation Details: Scripts, Toolchain, and Reproducibility

To improve reproducibility, this subsection summarizes how each encryption workflow was implemented and how measurements were collected. The experiment pipeline consists of (i) dataset preparation, (ii) payload generation with optional pre-encryption, (iii) LoRaWAN transmission using an identical radio configuration, and (iv) post-reception verification and logging. All scripts are available in a publicly accessible GitHub repository (https://github.com/ZaheenAfroz/Sertainty_Project) (accessed on 19 January 2026).

- Dataset preparation and payload formatting (common to all tests): A Python preprocessing script parses the smart meter dataset and extracts only the fields transmitted in this study (device identifier, firmware/hardware versions, temperature, millivolts, and a timestamp). Each row is compacted into a structured ASCII string and periodically dispatched to the end device to mimic real-time transmission. The LoRaWAN configuration held constant across all encryption settings. All the experiments used LoRaWAN DR3 (SF7; BW 125 kHz). To stay within the DR3 payload limit, the payload format was kept compact (parameter name initials + values), and the same end device, gateway, Network Server, and Application Server path were used for every run.
- Conventional encryption algorithms (ASCON-128, AES-256-GCM, SPECK, and XTEA): Encryption is performed in the host-side Python workflow immediately after constructing the compact ASCII payload and before transmission through the LoRa-E5 module. AES-256-GCM is implemented using a standard Python AEAD cryptography library interface (AESGCM), and ASCON-128 is implemented using a Python Ascon reference library configured with variant “Ascon-128”. SPECK and XTEA are implemented using self-contained reference implementations developed for this experimental workflow (SPECK-128/128 with 128-bit blocks and XTEA with 64-bit blocks), including PKCS#7-style padding to the respective block sizes.
  For AEAD schemes (AES-GCM and ASCON-128), a session key is generated once per experimental run, while a fresh nonce is generated per packet; the transmitted payload is formed as “nonce || ciphertext” (concatenation), encoded as hexadecimal, and sent using “AT+CMSGHEX”. For SPECK and XTEA, the plaintext is padded and encrypted in multi-block form, and the resulting ciphertext is transmitted as hexadecimal using “AT+CMSGHEX”. Encryption and decryption times are measured using “time.perf_counter()” and logged per packet along with the network timestamps (send_time, rx_time, meta_time, and ec2_publish). Correctness is verified by decrypting each received payload and comparing the recovered plaintext to the original compact payload string.
- Implementation note: The manuscript reports LoRaWAN latency at defined probe points (ED → gateway, gateway → NS, and NS → AS). The encryption/decryption time is recorded but not included in these network latency figures because encryption occurs before the packet enters LoRaWAN and decryption occurs after the packet exits the LoRaWAN path.
- Application Server logging and Network Server timestamps: The Application Server is an Ubuntu 22.04 AWS EC2 instance running a Mosquitto MQTT broker (v2.0) that receives uplink messages from the Everynet Network Server. A lightweight MQTT subscriber logs the received payloads and records “ec2_publish” timestamps. The gateway reception time (rx_time) and Network Server processing time (meta_time) are obtained from the Everynet JSON logs, using the timestamp definitions documented by Everynet.

**Figure 11:**
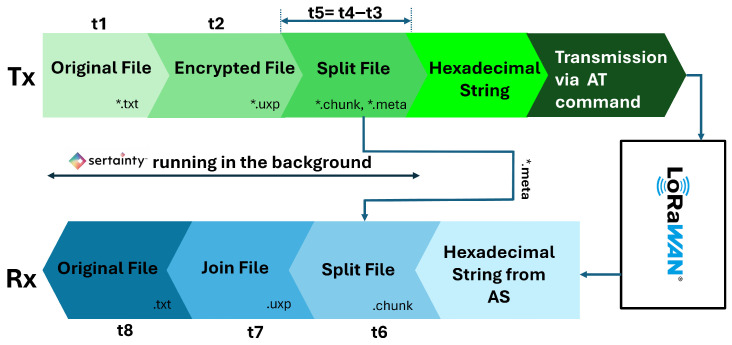
Monitoring different probe points in Sertainty test.

The Sertainty UXP experiments extended this methodology to a file-centric, policy-driven encryption workflow, as illustrated in the Sertainty flow diagram in Figure 11.

Instead of encrypting individual payload strings, an original text file was first protected by the Sertainty engine to produce an encrypted UXP Object (*.uxp). This file was then split into a set of fixed-size encrypted chunks (*.chunk) plus a metadata file (*.meta). Due to DR3 payload limits and duty cycle constraints, the chunk size was limited to 90 bytes, which resulted in 358 chunks for a single compacted dataset row and an effective inter-chunk period of about 15 s during stable operation. In parallel, a monitoring process observed the Sertainty pipeline folders and timestamped key events: the creation of the encrypted UXP file, generation of the first and last chunks, arrival of the metadata file that triggers the join operation, reconstruction of the joined UXP file, and the appearance of the final decrypted output. These filesystem-level timestamps were then combined with the usual LoRaWAN timestamps (rx_time, meta_time, and AS receipt time) to obtain a detailed timeline from the original file creation, through UXP protection, chunk transport over LoRaWAN, UXP reassembly, and final decryption back to plaintext. Figure 11 shows a simplified diagram of how different timestamps are measured during the Sertainty test.

In this study, latency is not reported as a single end-to-end value. Instead, latency is measured separately at different points of the LoRaWAN architecture: End Device–gateway, gateway–Network Server, and Network Server–Application Server. The encryption and decryption times are not included in final latency measurements. This is because encryption is performed before the data is sent into the LoRaWAN network, and decryption is performed after the payload is received from the network. For the other encryption algorithms, the encryption and decryption times did not change significantly across experiments. On the other hand, for Sertainty UXP, encryption and fragmentation (one additional step) are performed before transmission, and decryption is performed after reception in multiple steps. Therefore, the results describe LoRaWAN network latency and UXP processing time separately.

A confirmed uplink mode was used to support delivery confirmation and to explain possible latency outliers. In LoRaWAN, the ACK is sent as a downlink and can only be received by the end device in RX1 or RX2. If the ACK is not received, the same uplink may be retransmitted, which can create some high-latency points. Each payload was verified at the Application Server by comparing the sent payload with the received payload, and the next payload was transmitted only after a match was confirmed. The time gap between two uplinks was selected after several trial runs and was kept long enough for these steps to finish: (1) uplink transmission to the gateway, (2) processing at the Network Server, (3) sending and receiving the downlink ACK in RX1 or RX2, and (4) receiving and verifying the payload at the Application Server. This reduced the overlap between packets and reduced queue buildup. For each encryption setting, 100 packets were sent per run, and a run was accepted only when all 100 packets were received and verified. If any packet was not received, or if an ACK was not received after retry attempts, the run was stopped and repeated. Therefore, the reported latency results describe latency under successful delivery for a controlled baseline setup. Since the testbed used one end device and very low traffic at the gateway, missing packets and extreme outliers were less likely to occur. In future work, more end devices and larger distances will be added to provide more insight under higher load and weaker links.

## 3. Results

As mentioned in the Section 2, the original smart meter data has been encrypted with five encryption algorithms and tested over different distances. For each considered distance, 100 payloads have been sent for a certain encryption. The entire process has been logged from the start of transmission to the end of data retrieval after reception. The following subsections discuss the results of the tests that have been conducted. The Section 3.1 discusses results on raw utility data transmission over LoRaWAN. The subsequent sections report how the payload is processed and transmitted through the LoRaWAN probe points when different encryption techniques are applied.

Unlike the conventional lightweight ciphers evaluated in this study, Sertainty UXP is not designed as a drop-in payload encryption mechanism. Instead, it represents a data-centric security architecture in which encryption, identity binding, access policies, and lifecycle controls are embedded directly within the protected data object. As a result, comparisons with AES-256-GCM, ASCON-128, SPECK, and XTEA are intended to characterize performance trade-offs under identical LoRaWAN constraints, rather than to imply functional equivalence.

### 3.1. Raw Utility Data Analysis over LoRaWAN

Raw data refers to transmissions in which no additional encryption was applied prior to deployment in the LoRaWAN network. In this configuration, the compact 29-byte payload (obtained after preprocessing a single row of the smart meter dataset) was transmitted directly, allowing LoRaWAN to apply its fixed MAC layer overhead of approximately 13 bytes. This framing includes the MAC header, device address, frame control fields, frame counter, FPort, and a four-byte MIC, resulting in a total transmitted frame size of approximately 42 bytes. The decoded payload at the Application Server matched the original plaintext which confirms that no additional payload expansion occurs along the LoRaWAN path.

Across all distances, three latency components were extracted from synchronized timestamps and are reported as the mean ± standard deviation (n=100) in Table 2. The Gateway–NS and NS–AS segments remained comparatively stable across distances. Although moderate standard deviations are observed (e.g., 28.11 ms at 0 m and 33.02 ms at 200 m for Gateway–NS), median and interquartile range analysis indicates tightly clustered central behavior, suggesting that variability is primarily driven by occasional backend processing fluctuations rather than persistent instability.

In contrast, the End Device–Gateway segment shows the largest dispersion. At 0 m, the latency was 1376.85±47.42 ms, increasing to 2073.26±560.01 ms at 50 m. The elevated standard deviation at 50 m reflects sporadic high-latency transmission events, likely associated with retransmission or receive window timing effects. The median and IQR values confirm that the majority of packets remain tightly clustered. This behavior indicates that the observed dispersion is driven by limited, high-delay events rather than uniform degradation.

Beyond 100 m, the latency behavior changes markedly. At 100 m and 150 m, the End Device–Gateway segment shows tightly clustered distributions (1804.94±3.93 ms and 117.36±4.08 ms, respectively), indicating stable link conditions under clear, line-of-sight geometry. At 200 m, the mean latency is 159.72±303.23 ms. The large standard deviation indicates increased variability, and median-based analysis confirms that most transmissions remain closely grouped, with dispersion driven by isolated high-delay events.

The reduction in latency beyond 100 m is consistent with environmental conditions. The 0 m position was located inside a high-interference laboratory environment with structural attenuation, and the 50 m location experienced partial obstruction and non-line-of-sight propagation. In contrast, the 100 m and 150 m positions provided clear line-of-sight connectivity to the rooftop gateway, improving link quality and reducing retransmission frequency. This baseline behavior highlights the environmental sensitivity of the End Device–Gateway segment. It also serves as the reference condition for evaluating encryption-induced overhead.

**Figure 12:**
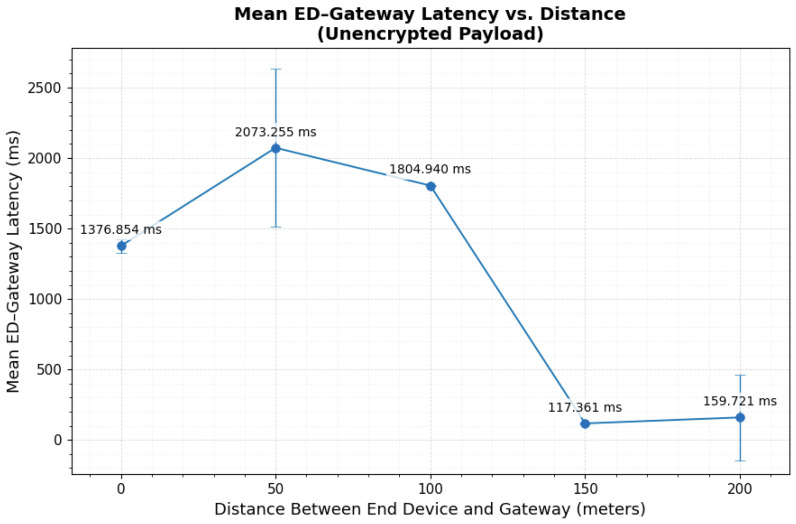
Average End Device–Gateway latency over tested distances.

Figure 12 presents the mean ED–Gateway latency as a function of distance for unencrypted transmission. Latency peaks at 50 m and 100 m and decreases significantly at 150 m and 200 m under improved line-of-sight conditions. Error bars denote ±1 standard deviation and illustrate the increased dispersion at 50 m and 200 m relative to other distances.

### 3.2. AES-256 GCM Encrypted Payload over LoRaWAN

AES-256-GCM expanded the 29-byte compact payload to 57 bytes (29-byte ciphertext + 12-byte nonce + 16-byte tag), producing an on-air frame of roughly 70 bytes once LoRaWAN’s 13-byte MAC overhead was added. At the Application Server, the payload consistently appeared at its expected encrypted size, confirming that no additional transformation occurred in transit.

Latency behavior largely followed the baseline trend, but dispersion became more visible in the End Device–Gateway segment. Table 3 reports the mean ± standard deviation (n=100). The Gateway–NS and NS–AS latencies remained stable across distances, and their central distributions stayed compact. For example, Gateway–NS medians remained near 81–89 ms with moderate spread (e.g., at 0 m: Q1–Q3 = 70.438–90.759 ms; at 200 m: Q1–Q3 = 70.561–92.840 ms). Similarly, NS–AS remained tightly clustered around ∼9–11 ms (e.g., at 0 m: Q1–Q3 = 9.994–10.889 ms; at 150 m: Q1–Q3 = 10.319–11.028 ms), indicating limited backend variability.

The End Device–Gateway latency showed strong distance dependence and the largest variability. At 0 m and 50 m, the mean ED–Gateway latency was high (1734.34±110.00 ms and 1691.37±84.55 ms, respectively). The interquartile ranges at these locations were small (0 m IQR = 5.979 ms with Q1–Q3 = 1756.307–1762.286 ms; 50 m IQR = 7.562 ms with Q1–Q3 = 1705.102–1712.663 ms), which indicates a consistent central latency level. These higher delays align with the same test geometry effects observed in the baseline, namely indoor interference at 0 m and partial obstruction at 50 m.

**Figure 13:**
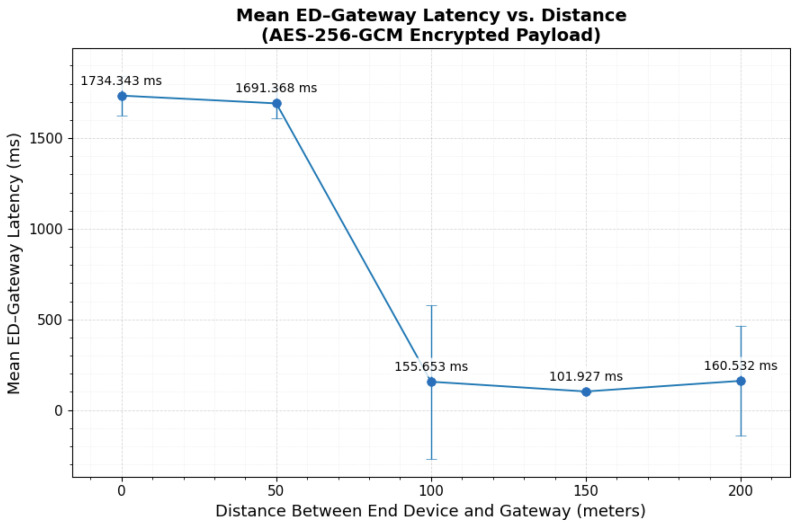
Average End Device-Gateway latency over tested distances using AES-256 GCM.

Beyond 100 m, ED–Gateway latency decreased sharply. At 150 m, the distribution was tightly clustered (101.93±3.66 ms; Q1–Q3 = 99.653–105.136 ms), which is consistent with clear line-of-sight conditions to the rooftop gateway. At 100 m and 200 m, the mean values were low (155.65±423.58 ms and 160.53±301.60 ms), but the standard deviations were large. The median and quartile statistics show that the typical latency remained stable at both distances (100 m: median = 95.670 ms, Q1–Q3 = 91.666–98.767 ms; 200 m: median = 130.591 ms, Q1–Q3 = 126.789–133.548 ms). This indicates that a small number of high-delay events inflated the mean and SD, while most packets stayed within a narrow central range. Figure 13 illustrates the overall distance trend and supports the conclusion that propagation conditions, rather than AES-256-GCM processing, dominate ED–Gateway latency behavior under the tested settings.

### 3.3. ASCON-128 Encrypted Payload over LoRaWAN

Under ASCON-128, the 29-byte compact payload expanded to approximately 61 bytes, resulting in an on-air frame of about 74 bytes after adding LoRaWAN’s 13-byte MAC overhead. The encrypted payload size remained consistent at the Application Server, confirming that no additional transformation occurred during transmission. The latency statistics in Table 4 show that the End Device–Gateway segment exhibited the largest distance-dependent variation. At 0 m and 50 m, the mean latencies were 1252.94±163.71 ms and 1680.33±97.46 ms, respectively. Despite moderate standard deviations, the central distributions remained tightly clustered (0 m Q1–Q3: 1229.416–1235.322 ms; 50 m Q1–Q3: 1686.184–1690.832 ms), indicating stable typical latency under indoor interference and partial obstruction. At 100 m, the mean latency increased to 1676.61±35.03 ms, with a narrow interquartile range (1676.760–1683.481 ms), reflecting consistent link behavior at that location.

**Figure 14:**
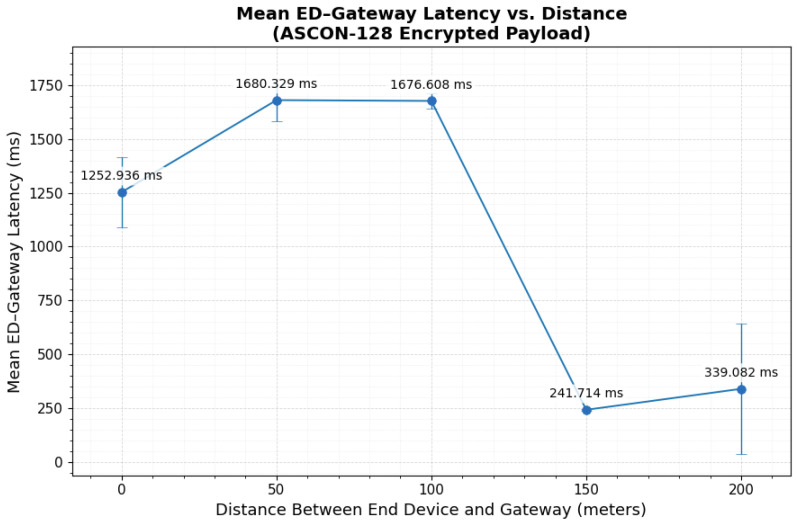
Average End Device–Gateway latency over tested distances using ASCON-128 encryption.

A substantial latency reduction was observed under clear line-of-sight conditions (Figure 14). At 150 m, the ED–Gateway delay decreased to 241.71±3.95 ms (Q1–Q3: 239.064–244.963 ms), indicating stable and tightly grouped transmissions. At 200 m, the mean latency was 339.08±301.81 ms. Although the standard deviation was large, the interquartile range remained narrow (305.686–312.524 ms), which confirms that variability was driven by a limited number of high-delay events rather than sustained degradation.

Gateway–NS and NS–AS segments remained stable across all distances. Gateway–NS latency varied within approximately 89–103 ms, while NS–AS remained tightly clustered around 9–10 ms. The interquartile ranges for both segments were moderate but consistent, indicating backend processing stability independent of encryption type. Overall, the latency trend under ASCON-128 mirrors the baseline behavior, and the observed variations are primarily influenced by propagation conditions rather than encryption overhead.

### 3.4. SPECK Encrypted Payload over LoRaWAN

SPECK encryption padded the 29-byte payload to 32 bytes to meet its 128-bit block size. The ciphertext was hex encoded to 64 bytes before LoRaWAN transmission, and the protocol added its fixed 13-byte MAC overhead. The encrypted payload size remained consistent at the Application Server. The latency results are summarized in Table 5. The End Device–Gateway segment showed strong distance dependence. At 0 m and 50 m, the mean latency was high (1760.60±214.19 ms and 1799.04±85.86 ms, respectively). The interquartile ranges indicate a concentrated central distribution despite the large means (0 m Q1–Q3: 1763.599–1836.738 ms; 50 m Q1–Q3: 1808.964–1814.231 ms). These values reflect indoor interference and partial obstruction, consistent with earlier experiments.

**Figure 15:**
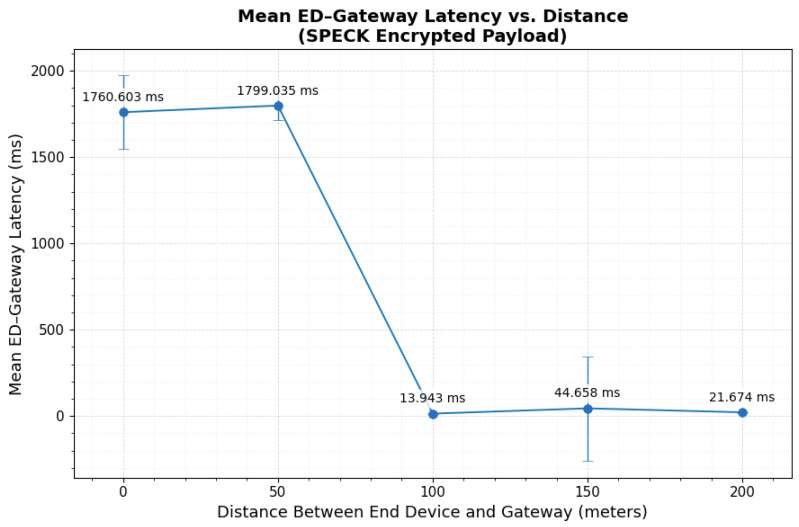
Average End Device–Gateway latency over tested distances using SPECK encryption.

At 100 m, latency decreased sharply to 13.94±4.84 ms (Q1–Q3: 11.042–16.440 ms), indicating stable and fast transmission under clear line-of-sight conditions. Figure 15 illustrates the mean ED–Gateway latency as a function of distance for SPECK encryption, with the error bars representing ±1 standard deviation. At 150 m, the mean was 44.66±301.45 ms, but the median remained 13.884 ms with a narrow interquartile range (10.794–17.458 ms). This shows that most packets experienced low delay, while a small number of high-delay events increased the mean and standard deviation. At 200 m, latency remained low (21.67±6.60 ms; Q1–Q3: 18.075–24.741 ms), confirming stable performance at extended distance. Gateway–NS and NS–AS segments remained stable across all distances. Gateway–NS latency ranged between 87–109 ms, and NS–AS latency remained within approximately 8.9–10.8 ms. The variations in these segments were moderate and did not show systematic distance dependence. Overall, SPECK achieved very low ED–Gateway latency at line-of-sight distances (100 m and 200 m), while high latency at 0 m and 50 m was driven by environmental conditions rather than encryption processing.

### 3.5. XTEA Encrypted Payload over LoRaWAN

XTEA expanded the 29-byte payload to 32 bytes through 8-byte block padding and, after hex encoding, produced a 64-byte transmission payload, followed by LoRaWAN’s fixed 13-byte MAC overhead. The encrypted payload size remained unchanged at the Application Server, confirming correct end-to-end delivery. The latency statistics are summarized in Table 6. The End Device–Gateway segment showed the strongest distance dependence. At 0 m and 50 m, the mean latency was high (1349.87±619.53 ms and 1777.44±136.72 ms, respectively). At 0 m, the interquartile range was wide (Q1–Q3: 1048.921–1266.442 ms), indicating substantial variability under indoor interference. At 50 m, the central distribution was tighter (Q1–Q3: 1800.141–1806.299 ms), despite the elevated mean.

A sharp reduction occurred under line-of-sight conditions. At 100 m, ED–Gateway latency dropped to 8.48±3.33 ms (Q1–Q3: 6.217–10.764 ms), representing the lowest mean latency observed among all tested configurations. At 150 m, the mean was 70.67±424.50 ms; however, the median remained 10.071 ms with a narrow interquartile range (6.822–13.338 ms), indicating that most packets experienced low delay while a small number of high-latency events increased the standard deviation. At 200 m, latency remained low (56.14±301.52 ms; Q1–Q3: 23.708–28.207 ms), again showing that dispersion was driven by isolated spikes rather than sustained degradation.

**Figure 16:**
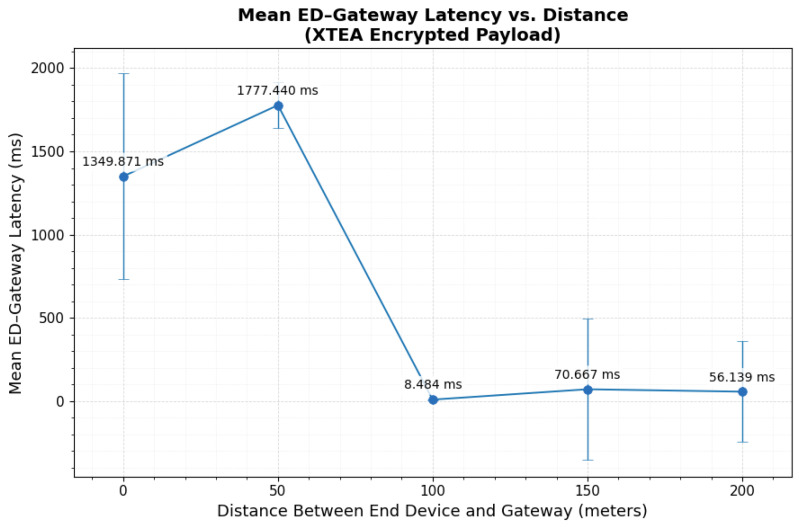
Average End Device–Gateway latency over tested distances using XTEA encryption.

Gateway–NS and NS–AS segments remained stable across all distances. Gateway–NS latency varied between 85–92 ms, and NS–AS latency remained within approximately 8.9–10.9 ms. The variations in these segments were moderate and showed no consistent distance dependence. Figure 16 illustrates the distance trend, showing the high latency at obstructed positions (0 m and 50 m) and a sharp decline at 100 m under clear line-of-sight conditions. Overall, the results indicate that ED–Gateway latency under XTEA is primarily governed by propagation conditions rather than encryption processing overhead.

### 3.6. Sertainty UXP Encrypted Payload over LoRaWAN

Sertainty UXP transformed the 29-byte plaintext into a 31.3 kB policy-enforced object. The object was divided into 358 chunks of approximately 90 bytes each, along with a 44-byte metadata file, to satisfy LoRaWAN DR3 payload limits. Each chunk was transmitted sequentially with an inter-packet interval of approximately 15 s. The measured non-LoRaWAN processing stages remained consistent with previous evaluation, totaling approximately 7.13 s (encryption, splitting, joining, and decryption combined).

The latency results are summarized in Table 7. The ED–Gateway segment exhibited a relatively stable mean across all distances, ranging from 634.94 ms (50 m) to 735.31 ms (150 m). At 50 m and 200 m, the variability was minimal (634.94±3.92 ms and 654.03±4.57 ms), with narrow interquartile ranges (50 m Q1–Q3: 631.591–637.683 ms; 200 m Q1–Q3: 650.470–657.476 ms). At 0 m and 100 m, the mean values were similar (720.13±402.15 ms and 713.03±462.24 ms), but the medians remained tightly clustered (0 m median: 665.836 ms; 100 m median: 641.580 ms), indicating that dispersion was influenced by a limited number of high-delay transmissions. At 150 m, the interquartile range widened (646.224–809.843 ms), reflecting moderate variability under that condition.

Gateway–NS latency remained within 82–99 ms across all distances, and NS–AS latency remained within 10.23–11.56 ms. These values are consistent with the trends observed in all previous tests and show no systematic distance dependence. Unlike lightweight block ciphers, UXP does not show a sharp reduction at line-of-sight distances. Instead, the ED–Gateway delay remains in a narrow band near 650–735 ms. This behavior is consistent with the deterministic chunking and fixed transmission schedule used in the UXP pipeline, where latency is dominated by structured packet sequencing rather than propagation effects.

### 3.7. Comparative Latency Summary Across All Algorithms

Across all tests, most of the latency change comes from the ED–Gateway segment. The Gateway–NS and NS–AS segments stay stable for every method and distance. Gateway–NS remains roughly in the 82–103 ms range, and NS–AS stays around 8.9–11.6 ms. This means the backend path is not the main driver of end-to-end changes. The main differences appear on the wireless hop between the end device and the gateway. Table 8 summarizes the radio link conditions at each testing point using RSSI and SNR values extracted from the Network Server uplink metadata. We report the mean ± standard deviation to show both the average link strength/quality and how much it fluctuated during the run. Overall, the 50–200 m points show relatively high and stable SNR (around 12–13 dB) with moderate RSSI variation, indicating consistently good reception. In contrast, the 0 m point shows lower average RSSI and SNR and larger variability, suggesting more unstable local propagation (e.g., multipath/interference near the setup). These metrics are provided to contextualize the channel environment under which the latency results were measured; because all payload types used the same LoRa PHY settings at each point, RSSI/SNR are not expected to be driven by the encryption method, but by the radio environment. The table also reports the number of uplinks (*n*), where *n* represents the successfully received uplinks for which RSSI/SNR metadata were available in the NS logs; small differences in *n* across distances reflect normal reception/logging variability rather than changes in the experimental procedure.

For all non-UXP methods, the ED–Gateway latency follows the same distance pattern. At 0 m and 50 m, the latency is high because of indoor interference and partial obstruction. For example, the unencrypted case is 1376.85 ms at 0 m and 2073.26 ms at 50 m. AES-256-GCM is 1734.34 ms at 0 m and 1691.37 ms at 50 m. ASCON-128 is 1252.94 ms at 0 m and 1680.33 ms at 50 m. SPECK and XTEA are also high at these points (SPECK: 1760.60–1799.04 ms at 0–50 m; XTEA: 1349.87–1777.44 ms at 0–50 m). Under clearer line of sight, latency drops sharply. AES-256-GCM reaches 101.93 ms at 150 m, and ASCON-128 reaches 241.71 ms at 150 m. The lightweight ciphers drop much lower: SPECK reaches 13.94 ms at 100 m and 21.67 ms at 200 m, while XTEA reaches 8.48 ms at 100 m. Some cases show large SD values at certain distances, but the median and IQR are usually narrow at the same points. This means most packets still arrive close to the typical value, and the large SD is caused by a small number of high-delay events.

Sertainty UXP shows a different ED–Gateway pattern. Instead of dropping sharply at line-of-sight distances, it stays in a narrow band across the full range. The mean ED–Gateway latency remains between 634.94 ms and 735.31 ms from 0–200 m. At 50 m and 200 m, the SD is very small (3.92 ms and 4.57 ms), and the IQR is also tight (about 6–7 ms), which shows very consistent timing. At 0 m and 100 m the SD is larger (402.15 ms and 462.24 ms), but the median and IQR still stay tight (for example, at 0 m the median is 665.84 ms with IQR 6.60 ms, and at 100 m the median is 641.58 ms with IQR 6.59 ms). This indicates that most chunks follow the same timing, but a small number of transmissions experience high delay and push the mean and SD upward. This behavior matches how UXP sends data. The payload is split into many fixed-size chunks, and chunks are sent in a fixed schedule with a fixed wait time between packets. So the ED–Gateway timing is mainly controlled by the transmission schedule, not by distance-based link improvement.

**Figure 17:**
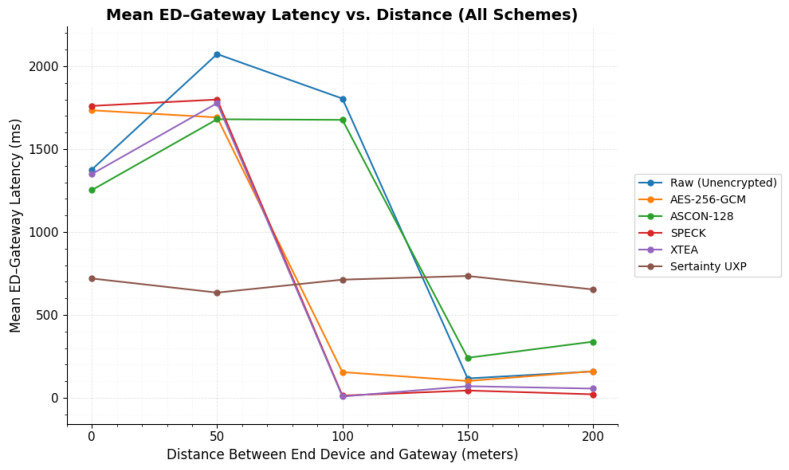
Mean ED–Gateway latency versus distance for all evaluated schemes (Raw, AES-256-GCM, ASCON-128, SPECK, XTEA, and Sertainty UXP).

The encryption and decryption timing results support the same conclusion (Shown in Table 9). For inline schemes, computation time is extremely small compared to network delay. SPECK and XTEA are on the order of 0.01–0.02 ms for encryption, and are similar for decryption. ASCON-128 is around 0.03–0.04 ms per operation, and AES-256-GCM is around 0.24 ms for encryption and 0.19 ms for decryption. These values are far below even the smallest wireless delays in the ED–Gateway results. UXP has a much larger processing pipeline (about 7.13 s total across encryption, split, join, and decryption), but this work is undertaken before sending and after receiving, so it does not change the per-chunk uplink timing. Overall, the combined plot in Figure 17 shows the same main message: the ED–Gateway latency is mostly controlled by the channel and the test environment for inline encryption methods, while UXP stays more consistent because its transmission is chunked and scheduled.

## 4. Conclusions

This research demonstrates that Sertainty’s Unbreakable Exchange Protocol (UXP) can be practically integrated into an LoRaWAN architecture to provide data-centric security in resource-constrained IoT deployments. Motivated by known weaknesses in LoRaWAN’s native security, particularly the use of AES in ECB mode during the join procedure, the work implemented UXP, which builds on AES-256-GCM and embeds identity-bound access policies directly into the protected data. Through comparative experiments with AES-256 GCM, ASCON-128, SPECK, XTEA, and UXP, the study quantified the impact of these schemes on payload size and latency, with end device-to-gateway delays ranging from roughly 720 ms to more than 1800 ms. While UXP incurred the largest processing and payload overhead, it maintained consistent end device-to-gateway latency across all tested distances and preserved end-to-end data integrity after chunking and reassembly, confirming its technical feasibility for LoRaWAN-based smart metering and similar utility applications. Overall, the results show that UXP offers substantially stronger confidentiality, integrity, and traceability than conventional LoRaWAN security at an acceptable performance cost, which motivates future work on lightweight UXP variants and the optimization of chunking strategies, and long-term field trials to evaluate scalability, energy efficiency, and operational robustness in real-world IoT settings.

## 5. Discussions

This study conducted a comprehensive real-world evaluation of five encryption schemes, which are the AES-256-GCM, ASCON-128, SPECK, XTEA, and Sertainty UXP. The implementation was performed within a unified LoRaWAN testbed to examine its effects on latency, payload overhead, and communication behavior. By synchronizing the timestamps at the end device, gateway, Network Server, and Application Server, the work provides a detailed decomposition of the LoRaWAN transmission pipeline. It also clarifies how encryption interacts with the physical environment. Across all traditional algorithms, the results show that environmental factors, rather than cryptographic processing, dominate uplink performance. Severe interference and partial obstruction at short distances (0–50 m) generated multi-second delays. In contrast, clear LOS at 100–150 m showed a reduction in the end device-to-gateway latency. This pattern aligns with prior LoRaWAN measurement studies and indicates that propagation conditions, rather than algorithmic complexity, are the primary source of latency variation in constrained IoT deployments.

In contrast to the other schemes, Sertainty UXP showed a distinct and highly consistent performance. Despite producing the largest payload (a 31.3 kB protected object divided into 358 chunks), UXP maintained a stable ED (end device)-to-gateway latency across all the distances tested. The values varied only between 634 and 735 ms. This behavior occurs because of UXP’s deterministic, chunk-based transmission pipeline, in which encrypted fragments of fixed size are dispatched according to duty cycle constraints. Because all cryptographic operations, including AES-256-GCM encryption, identity binding, metadata cloaking, and rule enforcement, occur before radio transmission and after reassembly, UXP effectively decouples its computational workload from channel conditions. The result is a minimally affected latency profile, unaffected by interference or LOS variability. The experiments also confirm the complete integrity of the reassembler and the feasibility of embedding the policy-driven, data-centric protection model of UXP within the severe payload and bandwidth constraints of LoRaWAN DR3.

Together, the comparative findings show that lightweight ciphers, such as SPECK and XTEA provide the lowest latency when propagation conditions are favorable. AEAD schemes such as AES-256-GCM and ASCON-128 provide stronger integrity and authenticity guarantees with only moderate overhead. UXP, although significantly heavier, demonstrates persistent identity, embedded policies, tamper awareness, and protection beyond network trust boundaries that are not achievable with conventional symmetric ciphers. These characteristics make UXP particularly suitable for applications in which data confidentiality, integrity, traceability, and survivability are more critical than minimal latency. Such applications include smart grids and advanced metering infrastructure, industrial control systems, healthcare IoT, distributed sensing, and other security-critical domains where data must remain protected even if the network infrastructure is compromised.

The study has several limitations. The experimental distances were limited to 0–200 m. However, this range was sufficient to expose LOS and interference effects. LoRaWAN typically operates over multiple-kilometer links, which warrant exploration in future works. UXP also operates as a proprietary “black box,” and the internal mechanisms of Sertainty’s KCL engine, cloaking processes, and access rule enforcement cannot be directly inspected. This limits the ability to analyze the causes behind its stable latency behavior. Power consumption was not evaluated, though it is a defining constraint for battery-powered IoT devices. In addition, the study used a single end device, leaving questions regarding scalability, collision dynamics, and duty cycle interactions in multi-device deployments. Finally, preprocessing operations such as chunking, joining, and timestamping were performed externally using Python scripts and the Sertainty Data Protector Utility rather than through embedded firmware. This may introduce overhead that would not be present in optimized implementations. Another limitation to consider is that several data modification steps occurred during the experiments. The process must be secured to prevent unauthorized access. Furthermore, because UXP’s internal protection composition and enforcement mechanisms are proprietary and its implementation source code is not publicly available, we rely on vendor technical documentation to describe the workflow and focus our evaluation on empirically measurable overheads observable during deployment.

Despite these constraints, the results have important implications for secure IoT design. The findings show that UXP can operate reliably within LoRaWAN’s strict DR3 payload and airtime limits, enabling persistent, data-centric protection for sensitive utility and industrial data. The demonstrated feasibility of combining UXP with LoRaWAN provides a foundation for future secure architectures in which the data object, rather than the communication channel, becomes the primary security boundary. Building on these insights, future work should examine multi-device scalability, energy profiling, long-range outdoor trials, embedded UXP integration, adaptive chunking strategies based on real-time link quality, and deeper security evaluations against replay attacks, metadata tampering, and packet loss.

In conclusion, this research confirms that advanced encryption schemes and data-centric protection frameworks can be integrated into LoRaWAN without compromising network functionality. Lightweight and AEAD ciphers remain appropriate for latency-sensitive applications. However, the deterministic behavior and strong embedded security guarantees of UXP make it a compelling model to protect critical IoT data. The results necessitate continued research into data-centric security approaches, the optimization of UXP for embedded systems, and large-scale empirical studies to support the next generation of secure, resilient IoT infrastructures.

## Figures and Tables

**Table 1:** Session keys in LoRaWAN.

Properties	AppSKey	NwkSKey
Purpose	End-to-end security between ED (end device) and AS (Application Server)	Authentication between ED (end device) and NS (Network Server)
Application	ED and AS	ED and NS
Key Distribution	OTAA (different for every join session). Static for ABP	OTAA (different for every join session). Static for ABP

**Table 2:** Unencrypted data latency reported as the mean ± standard deviation over different distances in the LoRaWAN network (n=100).

Latency (ms)	Distance (m)				
	0	50	100	150	200
ED–Gateway	1376.85±47.42	2073.26±560.01	1804.94±3.93	117.36±4.08	159.72±303.23
Gateway–NS	88.87±28.11	103.82±92.21	92.34±31.39	86.24±24.43	90.04±33.02
NS–AS	10.68±2.74	9.01±0.70	9.13±0.58	10.31±0.69	9.36±1.58

Latencies are given in milliseconds (ms) and reported as mean ± standard deviation (n=100).

**Table 3:** AES-256-GCM encrypted data latency reported as the mean ± standard deviation over different distances in the LoRaWAN network (n=100).

Latency (ms)	Distance (m)				
	0	50	100	150	200
ED–Gateway	1734.34±110.00	1691.37±84.55	155.65±423.58	101.93±3.66	160.53±301.60
Gateway–NS	90.83±30.82	93.17±26.46	94.30±36.51	95.40±56.46	89.49±29.18
NS–AS	10.58±1.07	9.22±2.13	10.90±0.63	10.73±0.75	9.24±1.90

Latencies are given in milliseconds (ms) and reported as mean ± standard deviation (n=100).

**Table 4:** ASCON-128 encrypted data latency reported as the mean ± standard deviation over different distances in the LoRaWAN network (n=100).

Latency (ms)	Distance (m)				
	0	50	100	150	200
ED–Gateway	1252.94±163.71	1680.33±97.46	1676.61±35.03	241.71±3.95	339.08±301.81
Gateway–NS	89.14±34.56	99.12±59.73	101.03±39.77	102.84±67.42	90.15±37.54
NS–AS	10.16±0.78	9.17±0.87	9.03±0.71	10.37±1.21	10.27±1.25

Latencies are given in milliseconds (ms) and reported as mean ± standard deviation (n=100).

**Table 5:** SPECK encrypted data latency reported as the mean ± standard deviation over different distances in the LoRaWAN network (n=100).

Latency (ms)	Distance (m)				
	0	50	100	150	200
ED–Gateway	1760.60±214.19	1799.04±85.86	13.94±4.84	44.66±301.45	21.67±6.60
Gateway–NS	92.27±47.54	109.49±59.76	88.71±27.24	87.38±30.44	89.35±41.59
NS–AS	9.40±4.24	8.94±0.58	9.88±1.02	10.81±0.68	10.54±0.90

Latencies are given in milliseconds (ms) and reported as mean ± standard deviation (n=100).

**Table 6:** XTEA encrypted data latency reported as the mean ± standard deviation over different distances in the LoRaWAN network (n=100).

Latency (ms)	Distance (m)				
	0	50	100	150	200
ED–Gateway	1349.87±619.53	1777.44±136.72	8.48±3.33	70.67±424.50	56.14±301.52
Gateway–NS	91.28±47.45	90.48±29.09	85.64±22.03	91.57±52.44	88.04±26.38
NS–AS	10.29±0.71	9.07±0.73	10.89±0.76	10.68±0.55	8.92±0.57

Latencies are given in milliseconds (ms) and reported as mean ± standard deviation (n=100).

**Table 7:** Sertainty UXP protected data latency reported as the mean ± standard deviation over different distances in the LoRaWAN network (n=100).

Latency (ms)	Distance (m)				
	0	50	100	150	200
ED–Gateway	720.13±402.15	634.94±3.92	713.03±462.24	735.31±360.65	654.03±4.57
Gateway–NS	82.84±25.84	93.11±47.78	93.00±53.66	99.45±54.76	82.21±19.59
NS–AS	11.56±5.85	10.32±0.62	10.23±0.53	10.25±0.59	10.28±0.71

Latencies are given in milliseconds (ms) and reported as mean ± standard deviation (n=100).

**Table 8:** LoRaWAN link-quality metrics (RSSI and SNR) reported as the mean ± standard deviation over different distances.

Metric	Distance (m)				
	0	50	100	150	200
RSSI (dBm)	−90.81±5.96	−80.77±4.52	−76.22±3.36	−71.18±4.01	−72.13±3.92
SNR (dB)	8.27±3.79	12.20±1.51	12.60±1.30	12.79±0.98	12.91±1.18
Uplinks (*n*)	277	285	292	287	278

RSSI is reported in dBm and SNR in dB as mean ± standard deviation, aggregated over all payload types at each testing point.

**Table 9:** Encryption and decryption time for different cryptographic algorithms.

Algorithm	Encryption Time (ms)	Decryption Time (ms)	Notes
AES-256	0.245	0.193	High security; higher processing time.
ASCON	0.039	0.030	Fast and secure; supports AEAD (NIST finalist).
SPECK	0.012	0.010	Ultra-fast; not widely standardized.
XTEA	0.019	0.015	Lightweight, simple design.

Execution times are given in milliseconds (ms).

## Data Availability

Data is unavailable due to privacy restriction. The authors will make the raw data supporting this article’s conclusions available upon request and permission granted by the sponsored corporation.
